# Experiences of smoking and tobacco use during pregnancy: A qualitative study protocol

**DOI:** 10.1371/journal.pone.0308781

**Published:** 2024-08-09

**Authors:** Maria Agràs-Guàrdia, Sara Martínez-Torres, Eva Satué, Ester Granado-Font, Meritxell Pallejà-Millán, Demetria Patricio, Miriam Leiva, Cristina Rey-Reñones, Francisco Martín-Luján

**Affiliations:** 1 Fundació Institut Universitari per a la recerca a l’Atenció Primària de Salut Jordi Gol i Gurina (IDIAPJGol), Barcelona, Spain; 2 Department of Primary Care Camp de Tarragona, Institut Català de la Salut, Primary Care Center Llibertat (Reus– 3), Reus, Spain; 3 ISAC Research Group, Institut Universitari d’Investigació en Atenció Primària Jordi Gol (IDIAP JGol), Barcelona, Spain; 4 Institut Universitari d’Investigació en Atenció Primària Jordi Gol (IDIAP Jordi Gol), Primary Healthcare Research Support Unit Camp de Tarragona, Reus, Spain; 5 Universitat Oberta de Catalunya (UOC), Barcelona, Spain; 6 School of Medicine and Health Sciences, Universitat Rovira i Virgili, Reus, Spain; 7 Department of Primary Care Camp de Tarragona, Institut Català de la Salut, Primary Care Center Horts de Miró (Reus– 4), Reus, Spain; 8 Institut Universitari d’Investigació en Atenció Primària Jordi Gol (IDIAP JGol), TICS-AP Research Group, Barcelona, Spain; 9 Department of Primary Care Camp de Tarragona, Institut Català de la Salut, Sexual and Reproductive Health Care Unit (ASSIR), Reus, Spain; Teesside University, UNITED KINGDOM OF GREAT BRITAIN AND NORTHERN IRELAND

## Abstract

Tobacco use during pregnancy is the main avoidable cause of morbidity and mortality both for pregnant women and their offspring. Between 12 and 22% of pregnant women in industrialized countries smoke during pregnancy, and 13% are unable to stop smoking. Pregnancy is considered an ideal opportunity to intervene and control tobacco use among smokers and their families. However, pregnant women experience barriers to quitting smoking, including social stigma and fear of being judged. Thus, it is necessary to develop interventions for smoking cessation adapted for pregnant women. This paper presents a qualitative study protocol to assess the barriers and facilitators of smoking cessation during pregnancy that female smokers encounter or perceive. It consists of a series of focus groups and individual interviews with female smokers who have been pregnant within the last five years. Participants will be recruited from the Sexual and Reproductive Health Care Services of the Camp de Tarragona. A group of 5–10 women who have been pregnant and tried to quit smoking over the last 5 years will be selected. The data will be collected by means of semistructured interviews. All interviews will be transcribed verbatim, coded and synthesized into categories and main themes. Thematic analysis will be conducted employing an iterative and reflexive approach. The results of this study will offer new perspectives on smoking interventions for pregnant women and enhance our comprehension of the main barriers to and facilitators of smoking cessation during pregnancy. This will contribute to the adaptation of the Tobbstop app, originally designed for the general public, to suit the needs of pregnant women. Consequently, the creation of targeted interventions will positively influence the health outcomes of both pregnant women and newborns.

**Trial registration:** Clinicaltrials.gov ID: NCT05222958. The trial was registered 3 February 2022, at https://clinicaltrials.gov/ct2/show/NCT05222958.

## Introduction

Smoking during pregnancy is a preventable risk factor associated with maternal and child morbidity and mortality [1]. Pregnancy is considered an ideal opportunity to intervene in and control tobacco consumption among smokers and their families [2]. However, 52.9% of women who smoke daily continue to smoke during pregnancy, and in the absence of intervention, they are likely to continue smoking [3].

Pregnant smokers face many barriers to and few facilitators of smoking cessation. Different variables, such as sociodemographic, relationship, social, smoking-related, pregnancy-related, health and psychological factors, can predict smoking cessation in pregnancy [4]. Partner support, willingness to change smoking habits, and the role of smoking in relationships are important factors. Recent studies have concluded that education to increase awareness and information about smoking during pregnancy are relevant. Moreover, providing access to effective interventions such as referrals to smoking cessation services, routine carbon monoxide screening, behavioral support, and pharmacotherapy could help pregnant women quit smoking [5]. These interventions effectively reduce tobacco consumption in the general population; however, pregnant smokers usually do not actively pursue them [6–8].

Lazarus and Folkman’s model defines the concept of stress by examining the intricate interaction between an individual and his or her environment. This model can be applied to investigate the role of psychological factors in smoking cessation [9]. According to their framework, anxiety levels depend on one’s ability to manage external demands, internal self-evaluations that exceed the individual’s available resources, and the strategies employed to cope with these stressors [10,11].

This conceptual framework is particularly relevant to our study because smoking cessation is widely recognized as a major source of stress. Although the majority of smokers older than 18 years of age express their desire to quit smoking (52% attempt to quit), only 6% successfully quit smoking after 12 months [12]. Previous research has highlighted the critical importance of stress-induced cravings, especially in people with high levels of nicotine dependence, who are at greatest risk of not quitting. Implementing stress coping programs has been shown to significantly improve the likelihood of successfully quitting smoking [13].

Behavioral support is the most effective smoking cessation intervention for pregnant smokers, but only a minority of pregnant smokers access it. Even though pregnant women are often aware of the negative effects of smoking on their fetus and themselves, they face many barriers that make it difficult to quit smoking. It is well known that the psychological and social environment also play important roles [13,14]. Consequently, additional qualitative studies are needed to analyze these barriers to identify strategies to help pregnant women quit smoking.

Digital interventions are accessible support interventions and alternatives to traditional support for smoking cessation. Moreover, digital interventions are preferred by women, as they note the importance of being informed to avoid cravings and continue abstinence [15].

Currently, only a few digital interventions for pregnant smokers have been developed, and little is known about the acceptability and usability of smartphone apps to aid in smoking cessation during pregnancy. In a study that aimed to determine the opinions of pregnant smokers about the use of an application for smoking cessation, it was concluded that this type of intervention could be feasible. The design of the application was considered a very important element, and pregnant women valued that the content was motivating, educational and personalized [16].

In a previous study, our research group addressed the effectiveness of an intervention based on gamification, the Tobbstop app, for reducing the prevalence of tobacco consumption in the young population [17,18]. Furthermore, a pilot study of a randomized controlled trial of pregnant smokers showed that pregnant app users had continuous abstinence until delivery; specifically, the prevalence of continuous abstinence until delivery was 57% in the intervention group versus 14% in the control group (p = 0.001) [19]. Building upon the findings of this pilot study, we intend to conduct a community-based research trial. This trial will be akin to the pilot study but with necessary adjustments to tailor the new Tobbstop app for pregnant women. The research group will conduct a study titled "Effectiveness of an App for Tobacco Cessation in Pregnant Smokers (TOBBGEST): randomized community trial". The Tobbstop app will be based on educational content, smoker support, recreational activities, games, entertainment features, and integrated social networking capabilities. This approach is based on the clinical practice guidelines for smoker support outlined by the Catalan Institute of Health and the Department of Health. The Department of Health is the regional institution responsible for planning health actions. The Catalan Institute of Health is the primary provider of public health services in Catalonia, ensuring healthcare for more than 80% of the population.

The original Tobbstop app was designed for the general public, and the intention was to adapt it to the needs of pregnant women. Considering the results of the study, messages and advice that are considered appropriate will be added, as well as information about the beneficial effects of smoking cessation on maternal, fetal and child health. Additionally, the app will offer audiovisual support in the form of mindfulness sessions and respiratory/muscle relaxation exercises [20].

We aim to determine the overall perceptions of female smokers regarding the facilitators of and barriers to smoking cessation during pregnancy through a qualitative research approach. The results of this new study will allow us to adapt the Tobbstop application to the needs of this population.

## Materials and methods

### Aims and objectives

The aim of this study is to identify the barriers to and facilitators of smoking cessation in pregnant smokers.

The specific objectives are as follows:

- To determine the difficulties or barriers encountered or perceived by pregnant women for smoking cessation.
- To determine the motivators or facilitators that help pregnant smokers stop smoking during pregnancy.
- To learn about the experiences of female smokers during pregnancy and tobacco addiction.

### Study design and setting

This study consists of a descriptive qualitative research design with a phenomenology approach and conversational techniques to determine the barriers to and facilitators of smoking cessation in pregnant women.

The Sexual and Reproductive Health Care Unit (Atenció a la salut sexual i reproductiva, ASSIR (Catalan acronym)) is a public Catalan Healthcare service for assistance and educational activities. ASSIR professionals include midwives, gynecologists-obstetricians, nurses, psychologists, and auxiliary administrative personnel who work in different locations in various consultations at health and staff center primary care. This study will be conducted at the ASSIR Centre of Reus (Camp de Tarragona; Spain).

### Recruitment

A group of 5–10 women who have smoked and received assistance at the ASSIR Centre for Pregnancy over the past five years and who have attempted to quit smoking will be recruited. The midwives within the ASSIR service, including a designated tobacco cessation expert, will be responsible for the recruitment process in their daily practice. The tobacco cessation expert is a professional with expertise in tobacco cessation and will be available for consultation in case of any uncertainties.

Inclusion criteria:

- Pregnant women who smoked during pregnancy within the past 5 years.
- Pregnant women who consent to participate in the study.

The exclusion criterion is language difficulties. Participants will be women who possess sufficient language proficiency to participate in an interview or focus group. Immigrant women will be asked if they feel capable of conversing in Catalan or Spanish; if their response is negative, they will be excluded. When individuals affirm proficiency but encounter comprehension difficulties during the interview, the research team will consider rejecting the interview.

Participants will be invited to join a focus group on a specific day and time. If they are unable to attend, they will be offered the option to participate in an interview at a time that suits their availability.

The health professionals responsible for recruiting pregnant women will explain the study and will be available to address any questions that may arise. Before conducting the in-depth interview or focus group, a member of the research team will provide participants with information about the study and will distribute an information sheet. Participants will have the opportunity to ask questions. Subsequently, each participant will be asked to sign the informed consent form.

We will employ thematic saturation as a criterion to determine the final number of interviewees [21]. To achieve information saturation, we will consider the key aspects outlined in S1 Appendix, the interview script, during the participant selection process. These considerations will include factors such as the number of children, the presence or absence of pregnancy-related difficulties, age, and previous experiences with tobacco cessation.

### Semistructured interviews and focus groups

A moderator experienced in qualitative research (the principal investigator or a member of the project team) and an observer will perform all the interviews or focus groups. Participants will have no previous contact with the research team before the sessions. Sessions will take place in a private space in the ASSIR center. A thematic scenario will be prepared (see S1 Appendix of the Supplementary Material for content details) that will be followed through the conversation. The interviews will continue until the point of relative saturation on the topic being discussed is reached, with a maximum duration of 90 minutes.

All interviews will be digitally audio-recorded and subject to the informed consent of the participants. In addition, an observer will take field notes during the session. Subsequently, an interviewer, who will record the data verbatim and anonymize any identifying data, will manually transcribe the participants’ information.

### Data management plan

Data from focus groups and individual interviews will be recorded, anonymized, analyzed and published. The records will be stored on a private secure informatic medium with password-protected access for exclusive use and validation by the researchers.

There is no intention to reuse these data, so they will be stored until the end of the main project and destroyed five years after publication of the final results.

### Data analysis

The data will be analyzed via reflective inductive thematic analysis to identify, interpret and report themes within the data [21].

First, interview recordings will be listened to and transcribed verbatim, including anonymized question answers/contributions from each group member. Next, before preparing the transcript for analysis, one person or, in case of doubt, multiple individuals will verify the accuracy of the transcription. Once the transcription of the content is approved, the research team will proceed with a semantic content analysis.

A thematic content analysis of the texts collected in the interviews or focus groups will be carried out by at least 2 members of the research team in the following way: (1) an initial reading of all the messages; (2) identification of relevant topics and text messages; (3) fragmentation of text into units of meaning; (4) coding of text by themes; and (5) creating categories based on Lazarus and Folkman’s model and the Cutrona model, grouping the codes; and (6) interpretation of the meanings of each category [10,11].

All the data for analysis will be analyzed and systematically organized using the qualitative analysis software ATLAS.ti (v5.14).

The final topics and categories (and possible subcategories) will be identified by induction, through analysis, deep reflection and discussion among the researchers. Preliminary conclusions will be presented in a meeting with the whole team and will give rise to an in-depth debate from which the conceptual framework will be created to develop an intervention to help pregnant women quit smoking. Furthermore, we will leverage strengths as a primary means of offering support whenever feasible. For instance, if women suggest that it would be beneficial to receive information about “mindfulness” or “nutrition” during the weaning process, these suggestions could be incorporated.

### Rigor and quality criteria

To guarantee the rigor and quality of the study, the rigor criteria suggested by Calderón will be followed: epistemological and methodological adequacy, relevance, validity and reflexivity [22]. The context and characteristics of the participants will be described in the research process. The messages obtained will be analyzed, and there will be a period of reflection that will be carried out by at least 2 members of the research team.

### Ethical considerations and declarations

This study will be conducted in agreement with the principles of the revised and updated Helsinki Declaration and Good Clinical Practice. The Clinical Research Ethics Committee of the Primary Care Research Institute (IDIAPJGol) (22/268-P; 25/01/2023) approved this protocol. Data confidentiality will be protected by the Spanish law governing the protection of personal data (Ley Orgánica de Protección de Datos de Carácter Personal y garantía de los derechos digitales; 03/2018, 5 December).

All participants will receive an information sheet from which they will be informed that their participation is anonymous, confidential and voluntary, as well as their right to change their mind (not to participate) at any time up to data verification. Verbal and written consent will be obtained from all participants to participate and be audio-recorded in the study.

## Discussion

The pregnancy and postpartum stages are periods of change and adaptation, during which important physiological changes occur. In addition, these periods are accompanied by worries and fears related to pregnancy, childbirth and motherhood [23].

While many women quit smoking when they become pregnant, many others continue smoking. These women may be conditioned by many factors that make it difficult for them to quit smoking [24]. Evidence has shown that the risk of mental health problems is greater in pregnant smokers than in nonpregnant smokers. Moreover, the occurrence of pandemics and sociodemographic and economic crises could lead to behavioral changes during pregnancy, leading to an increase in smoking prevalence [25,26]. Recent studies have reported that frustration in motherhood and having a partner who smokes are key barriers to achieving smoking abstinence during the postpartum period, while body weight maintenance during pregnancy and breastfeeding are facilitators of smoking abstinence [27]. In view of these factors, we believe that it is necessary to adapt the Tobbstop app based on the feelings and emotions experienced by pregnant women who attempt to quit smoking.

This study will provide information on barriers to and facilitators of the smoking cessation process in pregnant women, which will improve the content of digital interventions considering patient opinions. Moreover, in-depth patient interviews will contribute to a richer understanding of the health needs, lived experiences and barriers to quitting smoking during pregnancy among pregnant women.

Pregnant smokers frequently find themselves ensnared in a detrimental cycle. They turn to smoking as a coping mechanism to cope with stress and mental distress, even though they are aware of the harm it inflicts on both their unborn child and their own health. This leads to a cycle in which smoking exacerbates pregnant women’s feelings of guilt and vulnerability, all while facing societal pressure to quit. Thus, pressure to quit smoking intensifies addictions, suggesting that professionals should adjust their interventions to motivate patients and not stigmatize them [28]. The Tobbstop app was designed to support pregnant smokers during the quitting process but not to judge them.

This study has several limitations. First, there is the possibility of overrepresentation of groups that tend to spend time participating in focus groups, for example, unemployed individuals. Second, participants will be recruited through convenience sampling in a unique setting with geographic and cultural differences. Third, it must be considered that there are different profiles of pregnant women and that some of those selected may not choose a digital intervention to quit smoking. To mitigate these limitations, we will prioritize maximum variability and heterogeneity when selecting the participant sample.

In summary, this study aims to provide new information for adapting smoking cessation interventions for pregnant women and, consequently, for increasing the success of smoking cessation during pregnancy and the postpartum period.

## Supporting information

S1 Appendix Semistructured interview guide. This appendix aims to provide additional information about the study.
(DOCX)
