# Supplementary material for: Experiences of smoking and tobacco use during pregnancy: A qualitative study protocol
Source: PLoS One. 2024 Aug 9;19(8):e0308781. doi: 10.1371/journal.pone.0308781 (PMC11315274; doi:10.1371/journal.pone.0308781)
Supplement: S1 Appendix — This appendix aims to provide additional information about the study. (DOCX) [file pone.0308781.s001.docx]

**Appendix 1 - Semistructured interview guide**

**1.** **Introduction**

*Thank you for agreeing to participate in this interview. We are conducting these interviews to gain insight into the obstacles and challenges that female smokers encounter when attempting to quit smoking during pregnancy and how we can improve the supportive tools for smoking cessation during pregnancy.*

*Participation in this study is voluntary, and you may withdraw from the study at any time without explanation.*

*To protect your privacy, we are providing you with a signed confidentiality agreement.*

*I would like you to read it carefully and sign it.*

*The interview should take approximately one to one and a half hours, depending on how much information you would like to share.*

*With your permission, I would like to record the interview because I do not want to miss any of your comments.*

*All the responses will be confidential.*

*Do you have any questions?*

*This guide represents only the main themes to be discussed with the participants. The interviews will be restructured according to the participants’ interests, and the questions will be modified to specific situations.*

**2.** **General questions about pregnancy**

*With your permission, I will start with the interview.*

*Before we begin, it would be nice if you could tell me a little bit about your last pregnancy.*

- *Have you had difficulty getting pregnant?*
- *How many children do you have? How old are they? How did pregnancy affect your life in general?*

**3.** **Questions about pregnancy and smoking status**

- *Did you know that approximately 1 in 7 pregnant smokers fail to quit during pregnancy?*
- *Did you know that among its priority actions, the Catalan Health Plan aims to help pregnant women quit smoking?*
- *When did you start smoking? Do you remember why or what it was like?*
- *Are there people who smoke in your family, work or social environment? Who?*
- *What does smoking bring you (benefit, satisfaction...)?*
- *Have you ever considered quitting smoking? When?*
- *What are the main reasons that have lead you to consider quitting smoking?*
- Do you have information about or are you aware of the negative effects *of tobacco consumption (not only those related to health but also those related to beauty and the economy)? [If they only mention health-related effects, indicate that there are other effects as well and ask about them].*
- *If you thought about quitting smoking, what was your main difficulty in not being able to do it?*
- *Did you ask for help? Where? Did it help you?*
- *What do you think would have helped you quit smoking at that time?*
- Have you been affected by the opinions of others regarding smoking? *Did you feel uncomfortable in any situation?*
- *What were the main reasons for continuing to smoke?*

**4.** **Conclusion**

I believe that I have gathered all the relevant information regarding the study. Is there anything else?"

*In the next few days, we will provide you with a brief report about the information collected from your responses. Please, be sure that report’s information corresponds with your explanation.*

*Thank you very much for your time and the information you shared today.*
